# State Aid or State Advice

**Published:** 1920-01-17

**Authors:** 


### THE FUTURE OF THE BLIND.

### V.—State Aid or State Advice.

WHEN such a comparatively small class as one numbering 30,000 is affected, it is, to any who have been engaged for many years in charitable work, a little disappointing that one of the arguments given in favour of an Act of Parliament for the special benefit of that class should be that charitable organisation has tried and failed. Yet this failure was clearly in the mind of Mr. Stephen Walsh when, on November 25, he asked leave to introduce a Bill to provide for the technical education, employment, and maintenance of the blind, a copy of which Bill was issued a short time ago under the title of the Blind (Education, Employment, and Maintenance) Bill.

Mr. Walsh was Parliamentary Secretary to the Local Government Board in 1917, and was much associated with the formation and work of the Advisory Committee on the Welfare of the Blind. He is a Labour member, one who worked in mines from an early age, and knows personally how hardly the handicap of blindness bears upon the industry that he represents. He paid a compliment to many of the institutions for the welfare of the blind, and said that nothing is more admirable than the work they are carrying out. "But," he said, "there are, however, a vast number of organisations that really do not come within that description, and which, instead of doing their very best for the blind, too often utilise the blind, exploit them, and make a very good living out of their necessities and out of the charity of the wealthy."

With the advent of the National Institute for the Blind, the work of which body has recently been described in these columns, we had hoped that the future of the blind would, with the help of State advice, be assured, but that State aid would not be required. It is a pity that this cannot be so, as the fulfilment of the task, when placed in skilled hands, was but a matter of time and money. Here and elsewhere we have always been critical of the waste effort, chiefly the result of overlapping and muddle, that has characterised particularly the blind charities of the United Kingdom, but we hoped that the education of committees, in which we have played a humble part, was bringing about a better state of things sufficiently quickly to stave off Government interference.

### THE PROPOSED NEW ACT OF PARLIAMENT.

We understand that Mr. Walsh's Bill will not be proceeded with, and we need only briefly allude to its main features. Its object was to provide for the technical education of the blind by the establishment and equipment of technical schools or workshops, where necessary, or by contributions to existing institutions. Further, it provided for grants in respect of augmentation of wages and for expenses of living in hostels and institutions while the blind are under technical instruction; also for the employment and maintenance of blind persons away from workshops, and for the maintenance of blind persons incapacitated from earning their livelihood.

The Bill was a comprehensive one, and included for all those who require it the benefits of State aid, such aid to be given largely through local authorities and approved existing agencies.

One of the least satisfactory features of the Bill were the financial clauses, which, instead of stating maximum subsidies payable by the State, laid down minimum allowances for the training of the employable and the support of the unemployable blind.

We are informed that the Ministry of Health have in preparation a Bill which is more in accordance with the recommendations of the Advisory Committee on the Welfare of the Blind set up by the Local Government Board. This measure is expected to see the light early in 1920.

### BLIND CHILDREN.

Sir George Newman's recent report on the School Medical Service throws a light upon what we have said as to the large proportion of unemployable blind. He states that 20 per cent. of the blind population suffer also from mental deficiency, including epilepsy and serious nervous debility. Moreover, 12 per cent. of blind persons have not received any form of industrial training. The policy of the School Medical Service must aim at remedying these great defects, and its first object should clearly be prevention. We have already dwelt upon this point, but may mention that recently in the Sheffield Institution, out of 333 persons, no fewer than 136 were blind as a result of ophthalmia neonatorum.

The report states that at a specimen elementary school the curriculum includes general training in English, arithmetic, and other subjects, physical training, manual training, and musical training. The blind child must learn to walk with confidence, to swim, to play games. Those children who enter school unable to wash, dress, and feed themselves are at once taught those necessary actions. Manual training advances thereafter through various stages to industrial occupations. The importance of music can scarcely be exaggerated, for a great deal of the joy of life must depend on a cultivated ear.

There is only one secondary school for the blind—the Worcester College. Some 50 per cent. of pupils succeed in entering the universities. The careers usually followed are law, music, and the Church. A similar institution for girls, as mentioned in an earlier article, is to be founded by the National Institute at Chorley Wood.

There are "sight-saving classes" for partially blind children now established in many places, including twenty-one in London. The children are under oculist inspection, oral teaching replaces reading and writing to a great extent, and where writing is carried out it is undertaken in large script on blackboards or black linen rollers.

Blind children are now definitely classed in four great groups : (*a*) Totally blind ; (*b*) partially blind ; (*c*) myopes ; (*d*) mentally defective. All are being dealt with. The chief needs of the future are :

(1) Further provision for young blind children in special nursery schools.

(2) Increased attention to physical and musical training.

(3) Further provision of vocational training.

(4) Provision of higher type of craft teachers.

(5) Increased attention to after-care records.

(6) Encouragement of the local authorities in securing the early admission of all educable blind and myopic children to the school.

(7) Educational provision for mentally defective blind.

(8) Co-operation of local education authorities with the mental deficiency authorities to secure the notification, care, and custodial treatment of the ineducable blind.

**Previous articles appeared on October 18, 25, and November 15 and 28, p. 188.**

